# Supplementary material for: Effect of C-to-T transition at CpG sites on tumor suppressor genes in tumor development in cattle evaluated by somatic mutation analysis in enzootic bovine leukosis
Source: mSphere. 2024 Oct 15;9(11):e00216-24. doi: 10.1128/msphere.00216-24 (PMC11580432; doi:10.1128/msphere.00216-24)
Supplement: Supplemental figures — Fig. S1 and S2. [file msphere.00216-24-s0001.pdf]

Figure S1

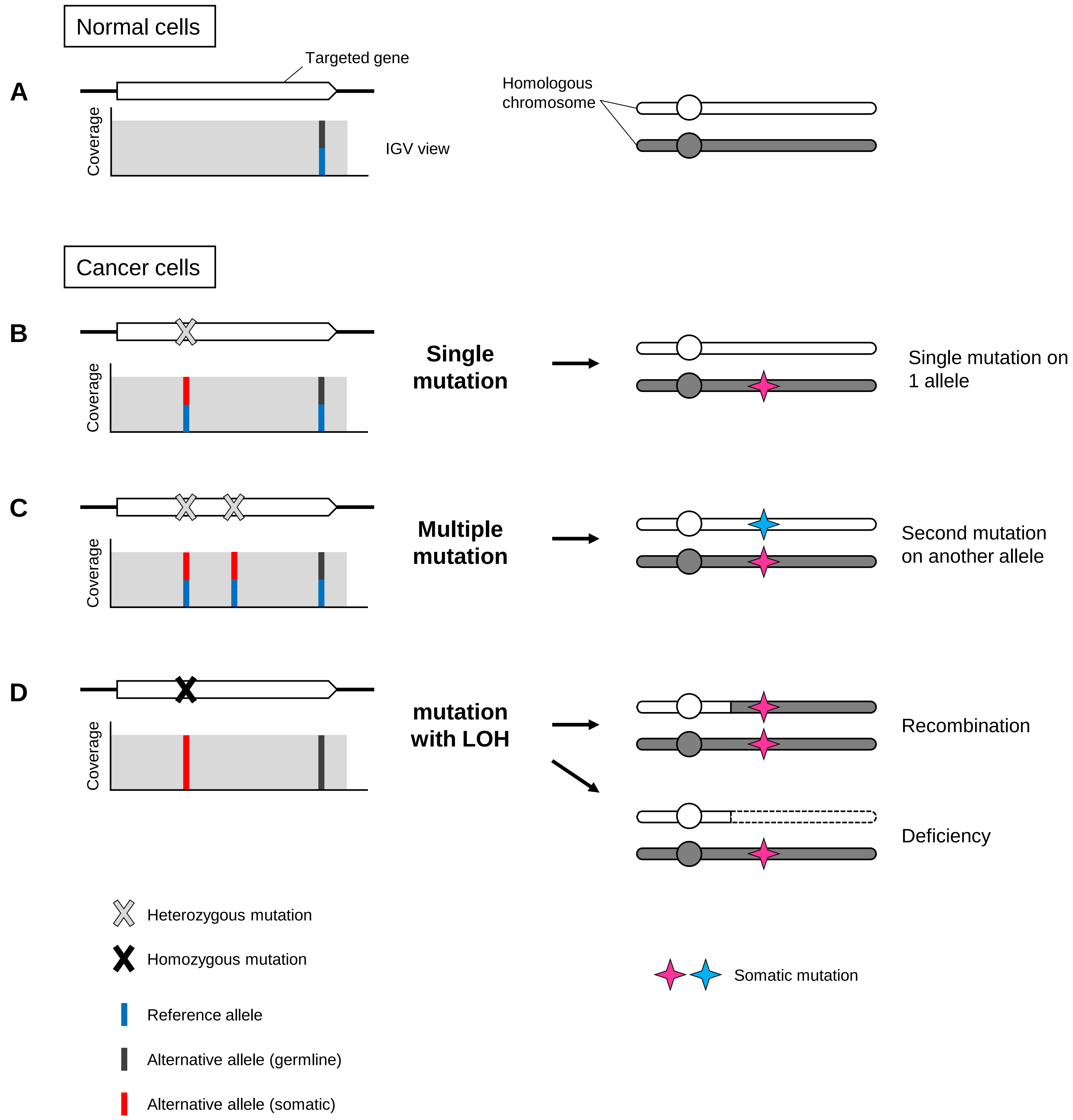

Supplemental figure 1

Diagram of data interpretation of somatic mutation analysis in this study. Images of the cording sequence (CDS) of targeted genes, read coverage in the IGV view, and homologous chromosomes are shown. (A) In normal cells, there are only germline mutations indicated in black on the targeted gene. (B) Single heterozygous somatic mutation indicated in red is observed in the CDS region of cancer cells. It is interpreted to be located on 1 allele of the homologous chromosome (pink). (C) Double somatic mutations are heterozygously observed in the same targeted gene. This result is interpreted to mean that the first mutation is located on one allele (pink), with the second mutation on another allele (sky blue). (D) Single homozygous somatic mutation is observed, which is interpreted as meaning that, following single heterozygous mutation on one allele, loss of heterozygosity (LOH) occurs by chromosomal recombination or deficiency.

Figure S2

A

COSMIC signatures	Proposed aetiology	
	Mutational process	Supported by
1	Spontaneous deamination of 5-methylcytosine (Aging)	mutational pattern
2	AID/APOBEC activity	experimental confirmation
3	HR deficiency	experimental confirmation
4	Tobacco smoking	experimental confirmation
5	Unknown (Aging / Tobacco smoking / NER deficiency)	age correlation / statistical association
6	MMR deficiency	statistical association; experimental studies
7	UV light exposure	experimental confirmation
8	HR deficiency / NER deficiency	statistical association
9	Polymerase eta somatic hypermutation	statistical association
10	POLE exonuclease domain mutation	experimental confirmation
11	Temozolomide chemotherapy / MMR deficiency + temozolomide	experimental studies
12	Unknown	unknown
13	AID/APOBEC activity	experimental confirmation
14	MMR deficiency + POLE mutation	experimental confirmation
15	MMR deficiency	statistical association; experimental studies
16	Unknown	unknown
17	Damage by ROS	statistical association
18	Damage by ROS	experimental confirmation
19	Unknown	unknown
20	MMR deficiency + POLD1 mutation	statistical association; experimental studies
21	MMR deficiency	statistical association; experimental studies

HR, homologous recombination; NER, nucleotide excision repair; MMR, mismatch repair; POLE, polymerase epsilon

B

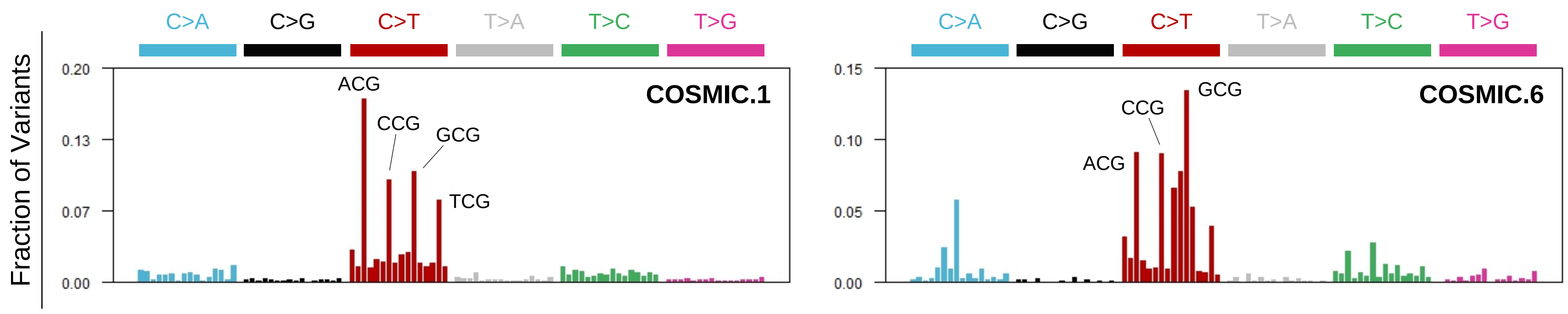

Supplemental figure 2

Information on known COSMIC signatures obtained from the database. (A) In COSMIC single base substitution (SBS) signatures 1–21, mutational process and supported evidence are listed based on the COSMIC website. (B) Mutation spectra of COSMIC SBS signatures 1 and 6 are drawn using MutSignatures R package and reference signatures datasets. Tri-nucleotide mutations at notable peaks in each signature are marked.
