# Supplementary material for: Effect of C-to-T transition at CpG sites on tumor suppressor genes in tumor development in cattle evaluated by somatic mutation analysis in enzootic bovine leukosis
Source: mSphere. 2024 Oct 15;9(11):e00216-24. doi: 10.1128/msphere.00216-24 (PMC11580432; doi:10.1128/msphere.00216-24)
Supplement: Table S1 — Basic information on clinical EBL cases evaluated in this study. [file msphere.00216-24-s0002.pdf]

Supplemental Table 1. Basic information on clinical EBL cases evaluated in this study

Case No.	ID	Age (month)	Sex[1)]	Breed[2)]	WBC (cells/μl)	Lym (cells/μl)	Cv blood	Cv tumor	Remarks
1	28-18	31	F	JB	13,900	6,000	-	-	case #18 in ref.19
2	24-14	43	F	HO	6,000	1,500	-	-	case #14 in ref.19
3	10-22	74	F	HO	13,100	7,900	-	-	case #22 in ref.19
4	45-7	67	F	HO	9,600	3,300	-	-	case #07 in ref.19
5	26-23	50	F	HO	10,200	4,700	-	-	case #23 in ref.19
6	29-36	67	F	HO	13,400	4,800	-	-	case #36 in ref.19
7	33-4	88	F	HO	6,700	2,200	-	-	case #04 in ref.19
8	53-10	181	F	JB	8,100	4,700	-	-	case #10 in ref.19
9	21-35	91	F	HO	13,100	3,400	-	-	case #35 in ref.19
10	27-24	25	M	JB	5,100	2,600	-	-	case #24 in ref.19
11	38-44	28	F	JB	4,600	1,800	-	-	case #44 in ref.19
12	40-6	81	F	HO	4,900	1,700	-	-	case #06 in ref.19
13	41-42	82	F	HO	7,200	2,700	-	-	case #42 in ref.19
14	44-39	82	F	HO	9,690	5,460	-	-	case #39 in ref.19
15	46-20	48	F	HO	25,800	2,000	-	-	case #20 in ref.19
16	50-9	83	F	HO	7,600	3,800	-	-	case #09 in ref.19
17	52-40	75	F	HO	8,300	1,770	-	-	case #40 in ref.19
18	34-38	84	F	HO	15,400	8,400	-	-	case #38 in ref.19
19	18-11	133	F	JB	30,700	9,800	-	-	case #11 in ref.19
20	20-17	127	F	JB	1,600	1,300	-	-	case #17 in ref.19
21	22-3	54	F	HO	5,000	2,100	-	-	case #03 in ref.19
22	30-19	78	F	HO	5,200	3,700	-	-	case #19 in ref.19
23	43-15	111	F	HO	41,500	12,100	-	-	case #15 in ref.19
24	32-37	118	F	HO	9,400	6,700	-	-	case #37 in ref.19
25	36-5	30	F	JB	6,700	2,400	-	-	case #05 in ref.19
26	39-41	136	F	JB	8,700	3,000	-	-	case #41 in ref.19
27	49-43	21	F	JB	5,130	1,670	-	-	case #43 in ref.19
28	EBL002	34	F	HO	-	-	0.09	1.00	ref.20
29	EBL024	17	F	JB	-	-	0.30	0.82	ref.20
30	EBL065	27	F	F1	-	-	0.11	0.52	ref.20
31	EBL070	29	M	JB	-	-	0.39	1.00	ref.20
32	EBL098	22	F	HO	-	-	0.10	0.79	ref.20
33	EBL221	28	M	JB	-	-	0.55	0.78	ref.20
34	EBL188	26	M	JB	-	-	0.31	0.95	ref.20
35	EBL119	133	F	HO	-	-	0.08	0.74	ref.20
36	EBL184	93	F	HO	-	-	0.11	1.00	ref.20

1) M, male; F, female

2) HO, Holstein Friesian (dairy cattle); JB, Japanese Black (beef cattle); F1, mix breed (HO×JB)

WBC, whole blood cells; Lym, lymphocytes; Cv, clonality value measured by BLV RAISING-CLOVA
