# Supplementary material for: Effect of C-to-T transition at CpG sites on tumor suppressor genes in tumor development in cattle evaluated by somatic mutation analysis in enzootic bovine leukosis
Source: mSphere. 2024 Oct 15;9(11):e00216-24. doi: 10.1128/msphere.00216-24 (PMC11580432; doi:10.1128/msphere.00216-24)
Supplement: Table S2 — Primers used for amplicon sequencing. [file msphere.00216-24-s0003.pdf]

Supplemental Table 2. Primers used for amplicon sequencing

Gene			Location	Sequences (5' → 3')	Product size	Multiplex set
TBL1XR1	1	F	90115086	ATGCTCAAGGCATTGTTCTTAGCTCTGT	8241	J
		R	90123327	CCACCAATAAAGAATGGAGCTGTTGCAC		
	2	F	90159639	TTCAGCTGGAGGGATCTTGAATTGGTTC	11823	K
		R	90171462	TCTCCCCACTTCAACCAAGACCTAGAAT		
	3	F	90171723	CAAGGAAAGCTGATGACAGACCAGAGAG	11914	L
		R	90183637	AACACTGTGCCACTGAGTGTAAAAAGGA		
	4	F	90191873	AGACCCAGTCTTAAAGAGAGAGGACCAC	11275	R
		R	90203148	CACCCTGAAAGCCTCTTGGTATCACATT		
	5	F	90201776	TCCATGCATCTGTGGTTTCAGCAGTATT	8321	N
		R	90210097	CAGTATCTCTTAAAGCAGCACGTTCGGA		
NRAS	1	F	28614522	TGTTCGACTTTTTATCACGGGAACGGAT	11053	G
		R	28625575	AATGAGGAAACTACTGGCTAGAGGAGCA		
EZH2	1	F	112078080	GCCACTCAGGGAGAAAAATACCTTCCAA	8267	F
		R	112086347	AAACAAGCCTCATCAAAACCTCCATCCT		
	2	F	112059203	GAAATGGAGAACCCCAGAAGAAGTCAGG	11234	G
		R	112070437	TACCTTCACAGGTGATTGTGATGCTTCC		
	3	F	112049746	TAACACGTGAAGGGCAATGTCAGGATAC	9484	A
		R	112059230	CCTGACTTCTTCTGGGGTTCTCCATTTC		
	4	F	112038557	AAACACCAGACTTAACGCTCAACAGACA	11216	I
		R	112049773	GTATCCTGACATTGCCCTTCACGTGTTA		
	5	F	112028761	CTAATTCAGAGTCTGGACGACCAAGCAG	10346	K
		R	112039107	TCGTTTCATACGCTTTTCTGTAGGCGAT		
	6	F	112023097	CATCTCCTGATGTGGTCACTCATTCCAC	5534	U
		R	112028631	GTAACTGAGATGGCACTGTCGTCAGAAA		
KMT2D	1	F	30757566	AGTGCAGAGCATATTACAGTGTTTGGCT	9893	L
		R	30767459	CTGCAGACATAACACACTCTGCTCCTAC		
	2	F	30761730	CCTTTCACCTCTCCAAGACTCCATTTCC	10586	N
		R	30772316	ATAACTGATCCCATTCCCCAGACCAGAT		
	3	F	30771219	TAAACACCCTTTAAATCCTGCCAGTCCC	11557	O
		R	30782776	AGGACTCTGCTAATCAGACTTCAGGTGT		
	4	F	30781488	GGGACAAGAAGGACATCTTCAATGAGCA	11126	P
		R	30792614	TCTGGAGAACTTCAAGAGACCTCTACCG		
	5	F	30790054	CCAGAGAGCAAACCTTATGGAGTCTTGG	10550	Q
		R	30800604	CTGGGTGACCAGCTGCAAATCATTTTAC		
KRAS	1	F	84747210	CCAGGAACCAGCATGTATCTTTGGAAGT	7901	F
		R	84755111	AGTATGCTGGCTTTAGGAACAGGTGAAC		
	2	F	84771071	TTGGCAAGTGAAGACTGTGAGGAAAAGT	11777	T
		R	84782848	CACCTGGGTAAAGAAGTGATGCTGATGT		
	3	F	84782821	ACATCAGCATCACTTCTTTACCCAGGTG	11294	I
		R	84794115	TTTAGGCTGTGATGGTGCCTTTAAGGAG		
	1	F	112279875	CCATCAATTTACCCAAGGCCAAACACAG	3512	M
		R	112283387	TCGTCTTCATTATCCTGGCACCTCAAAC		
	2	F	112299341	CAGGAGAGGAGGAAGAGAAAGAGACACA	8186	N
		R	112307527	TGAATATGGCAGGACCCCAAAACTTTCA		
	3	F	112306698	TGGAGATGAATGGCAAAACCTGCTTAGT	11112	O
		R	112317810	TTCATTCCATAAACACGCACTTAGGGCT		

EP300	4	F	112313499	TAATCTTGGCTTGTGCCCTTTGTCAGAT	11004	P
		R	112324503	TTGCTTGCCTCATTACCATTCAGTAGCA		
	5	F	112323546	CTTCACAGGAAGCAAGAATGGAGCCTAA	9050	Q
		R	112332596	ACAATAAAGGAGGCGATTACGAAACGGT		
	6	F	112330407	GCTCAGGCTTCTAAGTGGTCTTGGATTT	9085	R
		R	112339492	CTTTTCATTAGGCAACAGCAGCCTTAGC		
	7	F	112338018	GCCAGAAATACTGACTTCAGACCAGACC	9705	S
		R	112347723	GCTGGTTTTGAGGATTCAGTGAGCTAGT		
TET2	1	F	20036193	ACTCTGGAAGTCTGTCATAGCAACTCCT	4260	H
		R	20040453	CGAGAGTCCGTCATTCGGAGTTTAGTTC		
	2	F	19994792	ACTGAGACAAAGTAGCCAGAGGTGAGAT	3552	M
		R	19998344	CTCTTTGATGATTTGCCAGCTTGGTTCC		
	3	F	19931817	ACCAACACCAGCTCGAGATGTCTTAATG	10427	E
		R	19942244	CTATTAGGCAGGAATTCGCAGAAGGAGG		
	4	F	19913146	AAGAAACACCATGCAGGAGTCTGTGAAT	9347	F
		R	19922493	TAAGACTGTTGTTGTCCAGTGAGGTCAG		
	5	F	19894676	GTTACTCCATTCCTGACACTGGCTACAC	11429	G
		R	19906105	AAAGCATTTGCCCCATTTCACCTCATTC		
TNFAIP3	1	F	75739861	GGGGACACCAAGGCAATAAAAGGACTTA	10824	O
		R	75750685	CTTGAACGGGGATTTCTACCACCATCAA		
	2	F	75748250	AGCAGAGAGGCTGGTTTATTCTGGAAAC	9435	A
		R	75757685	TTGGCAAGGTGTTGATTGTTGAAACTGG		
B2M	1	F	103094337	CCACCAGGTAACGTCAGCTCCTTTTTAT	8755	B
		R	103103092	AGATCACAGCACCACCAAACTTATCTAAC		
	2	F	103102027	GGTATTTCTTCACAGGCTCTTCTGCCAT	8937	C
		R	103110964	AGCAGCCACCTAAGATGTTCATTCTCAC		
NOTCH1	1	F	103976741	GAAACAAGATTTAGGGCATCAAGCGTCG	5409	H
		R	103982150	AGGGGCCTAATTGGTGATTGGAAAAACT		
	2	F	103952668	TTTAGATAGAGCCAATGCCAGGCACGTA	8892	B
		R	103961560	GTAGGCAGGTCCCTGAAACAAAAGGTTG		
	3	F	103945505	AACACTTGTAGGTGTTGGTGAGGTCGAT	8879	C
		R	103954384	CTTGCAGATCAGGGTCTCAGGACTATGG		
	4	F	103937522	GGTTTTCTCATCTGTACAGCGGACATGC	10545	E
		R	103948067	GTCCATTCTCCCTTGGGGATTAGAAGCA		
	5	F	103933639	AGAACTTACCCCACTCACTATGTCACCA	9045	D
		R	103942684	CAAGATGGGGAAAGAGACCTGGGATTTC		
ATM	1	F	17842528	CCACTGAAGAAGATTCACCGCTAGTCTG	10417	L
		R	17852945	ATTTTTCTCCCCCTGCAATACCTCACAG		
	2	F	17851849	TAGGTACCTGCCTGTATGGTTCTTTGGA	9895	D
		R	17861744	TTCAGAGAACGTGCCAGATGATGGAATC		
	3	F	17864052	CACAGTCTTGGGGTTTATGGTGATGAGG	7241	N
		R	17871293	AAACTACTGAGTGGGAGTTTGTAAGGACA		
	4	F	17877642	ATGCCCTTCTACTTATCTCTGCTTGACC	7116	O
		R	17884758	ATGACTTCTCTCCCTTTTCATGCAACCA		
	5	F	17888675	TTGGCCATCAGGACATGCTTCTGTATTT	9352	P
		R	17898027	AGTGAGCTATGACTGTGCTAGACCTTGA		
	6	F	17897344	AACATGGTTAGCTGAAGTGTGATAGCCC	9826	Q
		R	17907170	AGTGTAAGCTGGAAGATTGGGTGAAAGG		
	7	F	17908087	TGCCTGTAGCAAGAAGTGGGTAATGAAG	10181	R
		R	17918268	AGCACCTCACATTTCCCCAGTATTTCAG		
	8	F	17915427	GTTCCTCAGTGTCCATCCCACAGTAAAG	7527	S

ATM	[illegible]				[illegible]	[illegible]
		R	17922954	TTTATTCCCCAAGAGAAGGAGAGGGTGT		
	9	F	17923784	TGGGACTTTTATATGGCAAGTGGCTTGT	9517	T
		R	17933301	CTTCCTTTTGTCTGGGATTGTCTCCCTC		
	10	F	17931396	GATCCACAACCCCTGCAAATTTGGATTC	9577	J
		R	17940973	ACCTGAATTATTCCTGCCTGACGAGTTG		
	11	F	17939736	GTCTAGCCCTCACCTTTCATTCAACCTC	8088	A
		R	17947824	AAGATCTCCAAAATGACTGTGCGTAGGG		
	12	F	17945469	TAGAGATCCTGCAGGCCCTAAAATCCAT	8065	B
		R	17953534	CCACTTTCTGGAGCGGAACAGTAATACA		
	13	F	17960654	CCACTGCTGCATCTAGCACAGTAAAATG	7533	C
		R	17968187	CAGGTACTGCCCATAATCCCATCATACC		
	14	F	17974174	AAAGGGAGTTTTGTCTCTTTCCCAGCAA	7154	D
		R	17981328	TAAATCTTCCTGGCCCCTTGTTCCTCTA		
	15	F	17986175	AAGAAGGCAGGCAGAGCATAGAACAAAT	11398	E
		R	17997573	GTCACTCAAGATTCCCATGAGAAGGTCC		
CD79A	1	F	51344192	ACTCGCTCTGTCTCTCCATCCTTATCTC	5520	B
		R	51349712	ACTGACCTGTGGCTGAAATGAAAGAACA		
TP53	1	F	27375293	GGCCCCAGTCTGGCCATCCTTCTAAT	13285	T
		R	27388578	CTGACTTTCCCCGCACTCTCCTCTCC		
CD79B	1	F	48131663	TAGGTGGAGGACTTTGAAGGACAAGAGG	4213	U
		R	48135876	AAGGGTATAAGTTGGTCTGGGGTGGAAG		
MYD88	1	F	11608904	CTCCCAATGTCAGTGTTTTCCCCTAGAC	5211	M
		R	11614115	CCAGGGATCTGTGATCTAGAATGGGGAT		
PIM1	1	F	11065620	AACATAAAAATCTGCCAGGGGATCTGGG	7639	A
		R	11073259	GGGTAGACATGAGTAGCGGTATTTGCAG		
BCL2	1	F	61581094	CTGCACAGGGTTGGAATTTATGGTCTCT	10119	S
		R	61591213	GAGTTAAGGCAAGTTCTGAGAAGACCCC		
	2	F	61392362	CTGAAAGACTCCACACCCTGATCCAATC	5422	C
		R	61397784	AACTCTGTGCTCTAATTCCAGGTTTGGG		
CREBBP	1	F	3168833	TCAACCATGCTGGAGAATCACACAATCA	7065	D
		R	3175898	TAAACACCTCCTGCCTTCTTATGCAGTG		
	2	F	3145134	GAAGCAGTTACCTGTAAGCAGAAGAGGG	7454	F
		R	3152588	ATGGCGTTAAGATAACTGGTGTCAGTGG		
	3	F	3111634	AGTGTGTGTACTGGGCACAACTTGTTTA	11791	E
		R	3123425	CTATACCTAGGGGTGACCCTCATCTGTG		
	4	F	3094675	CCTCATAAGAAGTCAGTTCGGGGGAAAC	9963	G
		R	3104638	TACTTCTCCACTCAGTACACTCAGCCTC		
	5	F	3087782	CCAAGGTGAACCGAAAGAGAGAGGAATC	7400	H
		R	3095182	GACAGACAGACGGACAGATGGGTATTTG		
	6	F	3076360	AAGATTTCTCAAGCATCCCTGTCACTCG	11858	I
		R	3088218	TGTTCCTTTGGATTGTGTTGTGCTTTGG		
	7	F	3064626	GAACACTCTGACACTGACCTCCAAAACA	8061	J
		R	3072687	TCATTCGGCTTGTTTACTTCTGCGGTAT		
	8	F	3052806	ACCTTTTGTTACCCAGATATTGCAGCCA	11847	K
		R	3064653	TGTTTTGGAGGTCAGTGTCAGAGTGTTC		
SOCS1	1	F	9911738	TTGGAAAACATCACCTCCTCCACTATGC	3040	M
		R	9914778	TATCCTAATGGGCTTGCCTGAAGAAACG		
	1	F	40415518	ACCGCAGCATTATGTCTTTCTGCTTAGT	3963	U
		R	40419481	ATAACAACGGCAAAGGTCGCTGTATGTA		
	2	F	40481333	ACATCCTGCCTTCTGCACTAATGATGAC	11727	P
		R	40493060	GGGTTCAGACCTGGAAGACCAGTATTTG		

CARD11	3	F	40492889	ATGGAAACAGTAAGTGGACCCAGTAGGA	9829	Q
		R	40502718	GGACAGTCCTGTGGGTTAAAGCAGATAC		
	4	F	40499092	AGGCTTTCTGAGGACTGTAACTGCTCTA	9172	R
		R	40508264	GTTACGGATGAGCTGGGTATTCATAGGC		
	5	F	40504522	ATTCCACAATGGGTATTGAGTGCCTGTT	11229	T
		R	40515751	ATCGGTGAAGCAGCAGGATTTAAGGAAA		
	6	F	40515724	TTTCCTTAAATCCTGCTGCTTCACCGAT	8343	S
		R	40524067	TCCCAGCTCATTAACATACACACAACCC		
PTEN	1	F	9462884	AAGGAAGAGCAGTGCTAATAACCGGAAC	4532	H
		R	9467416	TTTGCCTTCAAATGAGCACCATACATGC		
	2	F	9492424	GCTACCCATTTCTCCCTTCTTCAGCTTT	7004	J
		R	9499428	GAGGCATAAAGGCAGAGGTTTCTGAAGT		
	3	F	9529029	GTGTTGGCTGTGAGTGTTGTTTAGCTTC	10501	I
		R	9539530	TAAACATCTCAGGTCCTCTGCTCTGGAA		
	4	F	9550642	CACGGCTGTGAGAGACAAGAATAGAGTG	10729	K
		R	9561371	AGGTTTCCTCTGGTCCTGGTATGAAGAA		
	5	F	9555402	GTCCCTAGCAAACATCTGTCAACTCTCC	9351	L
		R	9564753	AGCTGGAGATGGTATATGGTCCAGAGTC		
