# Supplementary material for: Effect of C-to-T transition at CpG sites on tumor suppressor genes in tumor development in cattle evaluated by somatic mutation analysis in enzootic bovine leukosis
Source: mSphere. 2024 Oct 15;9(11):e00216-24. doi: 10.1128/msphere.00216-24 (PMC11580432; doi:10.1128/msphere.00216-24)
Supplement: Table S3 — Detail of somatic mutations identified in each EBL case. [file msphere.00216-24-s0004.pdf]

Supplemental Table 3. Detail of somatic mutations identified in each EBL case

Case No.	ID	Chr	Position	REF[1]	ALT[2]	Gene	VAF blood[3]	VAF tumor[3]	Fisher's exact test	Variant ID at the same position	Also called by[4]	Remarks
2	24-14	5	30793539	G	A	*KMT2D*	0.1462	0.2945	$p < 0.0001$	no	V	minor mutation
		19	27379331	C	T	*TP53*	0.2280	**0.9878**	$p < 0.0001$	no	V	LOH
3	10-22	19	27378376	C	T	*TP53*	-	**0.9535**	-	no	V, S, M	LOH
4	45-7	5	30792244	G	T	*KMT2D*	-	0.5028	-	rs447261496 (G>C)	S, M	-
		19	27380015	C	T	*TP53*	-	**0.9650**	-	no	S, M	LOH
5	26-23	5	84753447	G	A	*KRAS*	-	0.3241	-	no	V, S, M	-
		25	3061108	G	A	*CREBBP*	-	0.4867	-	no	V, S, M	-
		25	3147752	G	A	*CREBBP*	-	0.4769	-	no	V, S, M	-
6	29-36	11	103946432	C	T	*NOTCH1*	-	0.6622	-	no	V, S, M	-
		19	27378819	C	T	*TP53*	-	**0.9618**	-	no	S, M	LOH
7	33-4	19	27379331	C	T	*TP53*	-	0.4689	-	no	V, S, M	-
9	21-35	19	27378769	AAG	A	*TP53*	-	0.2428	-	no	V, M	-
		19	27378804	C	T	*TP53*	-	0.3233	-	no	V, S, M	-
10	27-24	19	27378778	C	A	*TP53*	-	**0.8251**	-	no	V, S, M	LOH
11	38-44	19	27378805	G	A	*TP53*	-	**0.8661**	-	no	S, M	LOH
12	40-6	19	27378808	G	A	*TP53*	-	**0.8936**	-	no	V, S, M	LOH
13	41-42	19	27379331	C	T	*TP53*	-	**0.9921**	-	no	V, S, M	LOH
14	44-39	19	27378842	G	T	*TP53*	-	**0.9713**	-	no	V, S, M	LOH
15	46-20	19	27378804	C	T	*TP53*	-	**0.9093**	-	no	V, S, M	LOH
16	50-9	19	27379331	C	T	*TP53*	-	0.6559	-	no	V, S, M	LOH*
17	52-40	19	27377694	G	A	*TP53*	-	0.4541	-	no	V, S, M	-
		19	27379338	C	G	*TP53*	-	0.4911	-	no	V, S, M	-
18	34-38	19	27378808	G	A	*TP53*	-	0.4743	-	no	V, S, M	-
19	18-11	19	27379319	T	C	*TP53*	-	0.2584	-	no	V, S, M	minor mutation
		19	27379331	C	T	*TP53*	-	0.2223	-	no	V, S, M	minor mutation
21	22-3	19	27378805	G	A	*TP53*	-	**0.7782**	-	no	V, S, M	LOH
		25	3078462	G	A	*CREBBP*	-	0.4442	-	no	V, S, M	-
		26	9466850	G	A	*PTEN*	-	0.4138	-	no	V, S, M	-
22	30-19	5	30777496	G	A	*KMT2D*	-	0.4460	-	no	V, S, M	-
		19	27378376	C	T	*TP53*	-	0.4553	-	no	V, S, M	-
		19	27379193	G	C	*TP53*	-	0.4060	-	no	V, S, M	-
23	43-15	25	40519575	G	A	*CARD11*	-	0.2381	-	no	S, M	minor mutation
25	36-5	19	27378376	C	T	*TP53*	-	**0.9996**	-	no	S	LOH
26	39-41	5	30796297	C	T	*KMT2D*	-	0.4813	-	no	V, S, M	-
		22	11611445	C	G	*MYD88*	-	0.5045	-	no	V, S, M	-
27	49-43	19	27378365	C	T	*TP53*	-	**0.9765**	-	no	V, S, M	LOH
28	EBL002	19	27378780	A	G	*TP53*	-	0.4907	-	rs450926493 (A>C)	V, S, M	-
29	EBL024	25	3085919	T	TG	*CREBBP*	-	**0.8078**	-	no	V, M	LOH
30	EBL065	5	84753447	G	T	*KRAS*	-	0.3456	-	no	V, S	-
		19	27379449	G	A	*TP53*	-	**0.7183**	-	no	V, S, M	LOH
		26	9538357	C	T	*PTEN*	-	0.1855	-	no	S, M	minor mutation
31	EBL070	19	27377679	G	A	*TP53*	-	**0.7273**	-	no	V, S, M	LOH
33	EBL221	19	27379400	G	A	*TP53*	0.2073	0.3434	$p < 0.0001$	no	V	-
		19	27380089	C	A	*TP53*	0.1486	0.2631	$p < 0.0001$	rs457482802 (C>T)	V	minor mutation
36	EBL184	19	27379974	C	T	*TP53*	-	**0.8714**	-	no	V, S, M	LOH

1) REF, reference allele

2) ALT, alternative allele

3) VAF, variant allele frequency; VAF more than 0.70 are shown in bold, and VAF from 0.30 (SNVs) or 0.20 (INDELs) to 0.70 are underlined.

4) V, VarScan2; S, Strelka2; M, Mutect2

* Although the tumor VAF is less than 0.70, this variant was exceptionally considered to be biallelic because presence of LOH in a wide range of *TP53* gene was suggested in this case.
