# Supplementary material for: Effect of C-to-T transition at CpG sites on tumor suppressor genes in tumor development in cattle evaluated by somatic mutation analysis in enzootic bovine leukosis
Source: mSphere. 2024 Oct 15;9(11):e00216-24. doi: 10.1128/msphere.00216-24 (PMC11580432; doi:10.1128/msphere.00216-24)
Supplement: Table S4 — Loss of heterozygosity of germline mutations in chromosome 19. [file msphere.00216-24-s0005.pdf]

Supplemental Table 4. Loss of heterozygosity of germline mutations in chromosome 19

Case No.	ID	Chr	Position	REF[1)]	ALT[2)]	Gene	VAF blood[3)]	VAF tumor[3)]	Variant ID
3	10-22	19	48132610	C	T	*CD79B*	0.5129	**0.9710**	rs134114145
		19	48133157	G	A	*CD79B*	0.4837	-	rs210668899
6	29-36	19	48132610	C	T	*CD79B*	0.5281	**0.9822**	rs134114145
11	38-44	19	27379196	C	T	*TP53*	0.5289	**0.9257**	rs133909661
		19	48132610	C	T	*CD79B*	0.4832	-	rs134114145
15	46-20	19	27377007	A	G	*TP53*	0.4822	**0.9503**	rs209064154
		19	27379184	C	T	*TP53*	0.5072	**0.9576**	rs456002482
		19	27380071	C	G	*TP53*	0.5010	**0.9621**	rs478425409
		19	48132610	C	T	*CD79B*	0.5064	**0.9614**	rs134114145
16	50-9	19	27377007	A	G	*TP53*	0.5046	**0.8147**	rs209064154
		19	27379184	C	T	*TP53*	0.5075	**0.8353**	rs456002482
		19	27380071	C	G	*TP53*	0.5012	**0.8217**	rs478425409
22	30-19	19	48132610	C	T	*CD79B*	0.4993	-	rs134114145
24	32-37	19	48132610	C	T	*CD79B*	0.5114	**0.9833**	rs134114145
27	49-43	19	27379196	C	T	*TP53*	0.4761	**0.9873**	rs133909661
31	EBL070	19	48133157	G	A	*CD79B*	0.4223	0.1936	rs210668899
36	EBL184	19	27377007	A	G	*TP53*	0.5937	**1.0000**	rs209064154
		19	27379184	C	T	*TP53*	0.5352	**1.0000**	rs456002482
		19	27380071	C	G	*TP53*	0.5518	**1.0000**	rs478425409

1) REF, reference allele

2) ALT, alternative allele

3) VAF, variant allele frequency; VAF more than 0.70 are shown in bold, and VAF from 0.30 (SNVs) or 0.20 (INDELs) to 0.70 are underlined.
